# The effect of interface anisotropy on demagnetization progress in perpendicularly oriented hard/soft exchange-coupled multilayers

**DOI:** 10.1038/s41598-017-03169-y

**Published:** 2017-06-27

**Authors:** Qian Zhao, Jun Chen, Jiaqi Wang, Xuefeng Zhang, Guoping Zhao, Qiang Ma

**Affiliations:** 1 Inner Mongolia Key Laboratory for Utilization of Bayan Obo Multi-Metallic Resources; Elected State Key Laboratory; Department of Applied Physics, College of Science, Inner Mongolia University of Science and Technology, Baotou, 014010 China; 2 College of Physics and Electronic Engineering, Sichuan Normal University, Chengdu, 610066 China

## Abstract

The demagnetization progress of various hard/soft multilayers with perpendicular crystalline anisotropy has been studied by a micromagnetic model, incorporating the effect of the interface anisotropy, which is evident on the nucleation field when the soft layer thickness is small. Both microscopic and macroscopic hysteresis loops as well as angular distributions for the magnetizations in the thickness direction have been calculated, taking into account of realistic values of the interface anisotropy. The formula for the nucleation field has been derived analytically, where the nucleation field increases linearly with the interface anisotropy for a wide thickness region. While the nucleation field could change by more than 90% due to the influence of the interface anisotropy, the interface anisotropy has no effect on the pinning field or the coercivity, but it has some slight influence on the angular distributions. On the other hand, positive interface anisotropy enhances the remanence and the energy products, whereas negative interface anisotropy deteriorates both of them. Comparison with the experimental data justifies our calculation, indicating that negative interface anisotropy should be avoided in the experiment.

## Introduction

Exchange coupled hard/soft magnetic systems, proposed formerly in 1991 by Kneller and Hawig^1^, have aroused a lot of attentions due to their specific properties and potential applications in permanent magnets^2–7^, magnetic recordings^8–13^ and other fields^14^. Exchange coupled magnetic systems, also called exchange-spring magnets, have a much larger energy product in theory^1, 15, 16^ than those of the conventional permanent magnets because the soft layer can provide large remanence while keeping the high coercivity of the hard phase. Among them, the hard/soft multilayers with hard and soft layers arranged alternatively in nanoscale have been a hot topic mainly due to their simplicity in modelling as well as the controllability in thickness-adjusted magnetic properties.

For hard/soft multilayers, there are many experimental^17–21^ and theoretical^22–27^ studies. In most calculations, however, only the volume crystalline anisotropy in every layer is taken into account, which is independent of the film thickness *t* ^28–33^, whereas experimental results indicate that the anisotropy in some magnetic films changes linearly along with 1/*t* ^34^. The crystalline anisotropy plays an important role in the magnetic reversal process^35–38^, which may be divided into two parts. One part is the volume crystalline anisotropy constant *K*_v_, and the other part is the surface crystalline anisotropy constant *K*_s_. The latter contribution to the anisotropy of the magnetic film can be expressed as 2*K*_s_/*t*. Surface anisotropy *K*_s_ includes the magnetocrystalline surface anisotropy caused by the lack of the nearest neighbor atom at the surface of the film^35^, and the magnetoelastic surface anisotropy induced by lattice mismatch^37^. For hard/soft multilayers, the crystalline anisotropy constant at the interface has to be considered also, which is the sum of the two related surface anisotropy constants^36, 37^. Experimental results show that the surface and interface anisotropy constants of the transition metals vary with the material, the lattice orientation and the interface character, which may be positive, negative and zero^37, 38^.

Recently, Pellicelli *et al*.^26^ have studied the effects of the positive crystalline anisotropies at the surfaces and the interface on the demagnetization progress in an exchange-coupled bilayer system through a continuous micromagnetic model. Their results indicate that the soft surface anisotropy has an evident effect on the nucleation field, whilst the hard surface anisotropy has appreciable influence on the coercivity when the corresponding layer thickness is small. The impact of the interface anisotropy, on the other hand, is in between.

**Figure 1:**
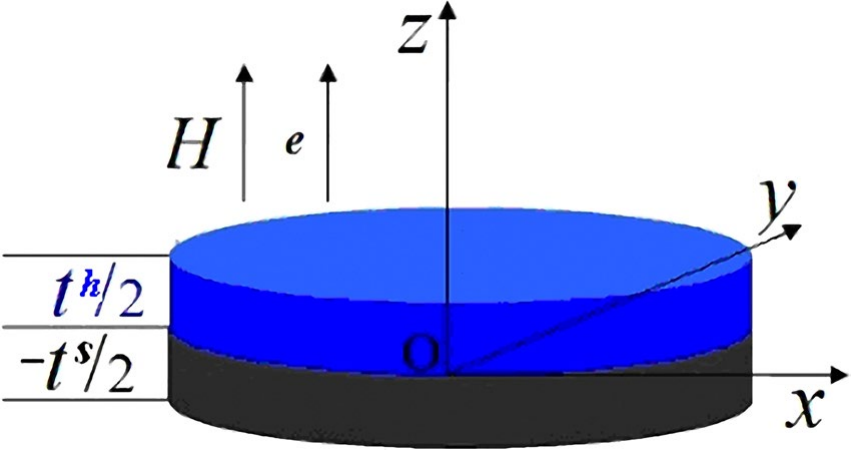
The basic scheme for a hard/soft multilayer calculated in this paper. Due to the symmetry, only a simplification of the symmetrical multilayer from −*t* ^*s*^/2 to *t* ^*h*^/2 is illustrated in the figure.

For a hard/soft multilayer, the interface anisotropy is more important than the surface anisotropy because there are multi interfaces against only two surfaces. In this paper, the influence of both positive and negative interface anisotropies on the demagnetization process of a hard/soft multilayer system with a perpendicularly oriented anisotropy shown in Fig. 1 is studied. The nucleation field is found to be linearly related to the interface anisotropy, where the formula for the nucleation field is derived analytically. Both macroscopic and microscopic hysteresis loops have been calculated numerically, with angular distributions for the magnetizations and energy products given. Comparison of our results with experimental data indicates that the interface anisotropy should be taken into account in calculating nucleation fields and hysteresis loops.

## Methods

Our calculation is based on a multilayer system, where hard/soft magnetic layers are arranged alternatively. Due to the symmetry of the system, the calculation can be performed only for a double-layer system shown Fig. 1, assuming that the interface anisotropy constant varies from −1 erg/cm^2^ to 1 erg/cm^2^ ^37, 38^. The *z*-axis and the easy axes of both phases are supposed to be perpendicular to the film plane, with the magnetic field applied parallel to the *z*-axis. For simplicity the film is assumed to be infinitely large in the *x*-*y* plane, indicating that the problem has a one-dimensional character^23–26, 28–33^.

According to Brown^39^, the total magnetic energy per unit area can be expressed as:

1 $${\int }_{0}^{\tfrac{{t}^{h}}{2}}{F}^{h}dz+{\int }_{-\frac{{t}^{s}}{2}}^{0}{F}^{s}dz+{K}^{int}\,{\sin }^{2}\,{\theta }^{0},$$

where ${F}^{i}={A}^{i}{(\frac{d\theta }{dz})}^{2}+{K}^{i}\,{\sin }^{2}\,\theta -H{M}_{S}^{i}\,\cos \,\theta +2\pi {M}_{S}^{i\,2}\,{\cos }^{2}\,\theta ,$ (*i* = *h*, *s*), and *i* = *h*, *s* for the bottom soft and top hard layers, respectively. *K* ^*i*^ and *K* ^*int*^ are the volume and interface anisotropy constants respectively. ${M}_{S}^{i}$ is the spontaneous magnetization while *θ* is the angle between the magnetization ${M}_{S}^{i}$ and the applied field *H* and *θ* ^0^ is the specific angle at the interface. *A* ^*i*^ and *t* ^*i*^ denote the exchange energy constant and the value for the thickness of the magnetic layers respectively.

The variation of the total energy density leads to two Euler-Lagrange equations,

2 $$2{A}^{h}\frac{{{\rm{d}}}^{2}\,\theta }{{\rm{d}}\,{z}^{2}}-(2{J}^{h}\,\cos \,\theta +{M}_{S}^{h}H)\,\sin \,\theta =0,\,0\le z\le {t}^{h}/2,$$

3 $$2{A}^{s}\frac{{{\rm{d}}}^{2}\,\theta }{{\rm{d}}\,{z}^{2}}-(2{J}^{s}\,\cos \,\theta +{M}_{S}^{s}H)\,\sin \,\theta =0,\,-{t}^{s}/2\le z\le 0,$$

where ${J}^{i}={K}^{i}-2\pi {M}_{S}^{i2}$ are the effective volume anisotropy constants. The above two euqations are coupled by the following interface equation^26, 28–33^:

4 $${{A}^{h}\frac{{\rm{d}}\theta }{{\rm{d}}z}|}_{{0}^{+}}={{A}^{s}\frac{{\rm{d}}\theta }{{\rm{d}}z}|}_{{0}^{-}}+{K}^{int}\,\sin \,{\theta }^{0}\,\cos \,{\theta }^{0},$$

with the following boundary conditions^26, 28–33^:

5 $${{A}^{h}\frac{{\rm{d}}\theta }{{\rm{d}}z}|}_{\frac{{t}^{h}}{2}}=0,\,{{A}^{s}\frac{{\rm{d}}\theta }{{\rm{d}}z}|}_{-\frac{{t}^{s}}{2}}=0.$$

The specific angles at the surfaces and interface are defined as:

6 $${\theta |}_{z=\frac{{t}^{h}}{2}}={\theta }^{h},\,{\theta |}_{z=\frac{-{t}^{s}}{2}}={\theta }^{s},\,{\theta |}_{z=0}={\theta }^{0}={\theta }^{{0}^{-}}={\theta }^{{0}^{+}}.$$

Solving Eqs (2) and (3), we obtain the angular distribution in the hard and soft phases as two elliptical integrals:

7 $${\int }_{{\theta }^{h}}^{\theta }\frac{{\rm{d}}\,\theta }{\sqrt{{J}^{h}({\sin }^{2}\,\theta -{\sin }^{2}\,{\theta }^{h})-H{M}_{S}^{h}(\cos \,\theta -\,\cos \,{\theta }^{h})}}=\frac{{t}^{h}/2-z}{\sqrt{{A}^{h}}},$$

8 $${\int }_{\theta }^{{\theta }^{s}}\frac{d\theta }{\sqrt{{J}^{s}({\sin }^{2}\,\theta -{\sin }^{2}\,{\theta }^{s})-H{M}_{S}^{s}(\cos \,\theta -\,\cos \,{\theta }^{s})}}=\frac{{t}^{s}/2+z}{\sqrt{{A}^{s}}}.$$

On the other hand, the interface constraint Eq. (4) could be rewritten as:

9 $$\begin{array}{c}\sqrt{{A}^{s}}\sqrt{{J}^{s}({\sin }^{2}\,{\theta }^{0}-{\sin }^{2}\,{\theta }^{s})-H{M}_{S}^{s}(\cos \,{\theta }^{0}-\,\cos \,{\theta }^{s})}\\ \quad =\,\sqrt{{A}^{h}}\sqrt{{J}^{h}({\sin }^{2}\,{\theta }^{0}-{\sin }^{2}\,{\theta }^{h})-H{M}_{S}^{h}(\cos \,{\theta }^{0}-\,\cos \,{\theta }^{h})}+{K}^{int}\,\sin \,{\theta }^{0}\,\cos \,{\theta }^{0}.\end{array}$$

Eqs (7)–(9) form the basis of our calculation. In this paper, we calculate the magnetic properties for various multilayers, with the intrinsic parameters given in Table 1.

**Table 1:** Intrinsic magnetic properties for various magnetic materials.

Materials	*A* (erg/cm)	*K* (erg/cm^3^)	*Ms* (emu/cm^3^)	Δ (nm)
α-Fe	2.50 × 10^−6^	4.60 × 10^5^	1.71 × 10^3^	73.20
Fe_65_Co_35_	1.67 × 10^−6^	1.00 × 10^2^	1.93 × 10^3^	4.06 × 10^3^
Nd_2_Fe_14_B	7.70 × 10^−7^	4.30 × 10^7^	1.28 × 10^3^	4.20
FePt	1.25 × 10^−6^	2.50 × 10^7^	5.00 × 10^2^	7.02
SmCo_5_	1.20 × 10^−6^	5.00 × 10^7^	5.50 × 10^2^	4.87

*A*, *K*, *Ms* and Δ denote the exchange constant, the volume anisotropy constant, the spontaneous magnetization and the Bloch wall width respectively.

## Results

### Nucleation fields

The formula for the nucleation field in this work can be obtained in a similar way to that for a hard/soft multilayer ignoring the interface anisotropy, as has been done in refs 23–25, 28–33. In short, linearization of the Eqs (7) and (8), coupled by the interface constraint Eq. (9), yields the following implicit equation for the nucleation field:

10 $$\begin{array}{c}\sqrt{{A}^{s}(-{J}^{s}+{H}_{N}{M}_{S}^{s}/2)}\,\tan \,(\frac{{t}^{s}}{2}\sqrt{-\frac{{J}^{s}}{{A}^{s}}+\frac{{H}_{N}{M}_{S}^{s}}{2{A}^{s}}})\\ \quad =\sqrt{{A}^{h}({J}^{h}-{H}_{N}{M}_{S}^{h}/2)}\,\tanh \,(\frac{{t}^{h}}{2}\sqrt{\frac{{J}^{h}}{{A}^{h}}-\frac{{H}_{N}{M}_{S}^{h}}{2{A}^{h}}})+{K}^{int},\end{array}$$

where *H*_*N*_ = −*H*, denoting the nucleation field for a hard/soft multilayer. The above formula is consistent with that derived by Zhao *et al*.^30, 31^ and Pellicelli *et al*.^26^ for the nucleation field at the case *K* ^*int*^ = 0. On the other hand, when both the soft and hard layers are thin enough, $\tan \,(\frac{{t}^{s}}{2}\sqrt{-\frac{{J}^{s}}{{A}^{s}}+\frac{{H}_{N}{M}_{S}^{s}}{2{A}^{s}}})$ and $\tanh \,(\frac{{t}^{h}}{2}\sqrt{\frac{{J}^{h}}{{A}^{h}}-\frac{{H}_{N}{M}_{S}^{h}}{2{A}^{h}}})$ can be replaced by $\frac{{t}^{s}}{2}\sqrt{-\frac{{J}^{s}}{{A}^{s}}+\frac{{H}_{N}{M}_{S}^{s}}{2{A}^{s}}}$ and $\frac{{t}^{h}}{2}\sqrt{\frac{{J}^{h}}{{A}^{h}}-\frac{{H}_{N}{M}_{S}^{h}}{2{A}^{h}}}$ respectively. As a result, Eq. (10) can be simplified as ${H}_{N}=\frac{2[({J}^{h}{t}^{h}+{J}^{s}{t}^{s})/2+{K}^{int}]}{({M}_{S}^{h}{t}^{h}+{M}_{S}^{s}{t}^{s})/2}$. This formula indicates that the whole system responds to the applied field coherently, where the nucleation field is given by the mean anisotropy divided by the mean spontaneous magnetizations of the system, with the thickness of the layer taken into account as the weight.

**Figure 2:**
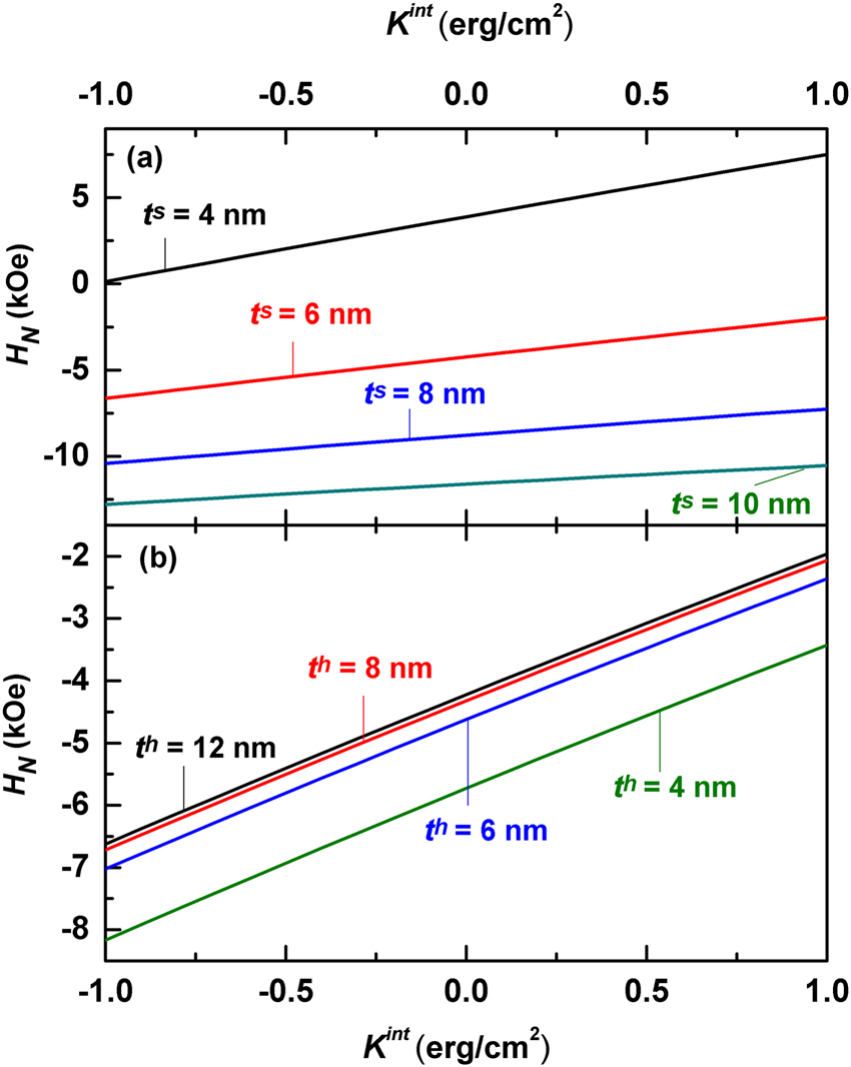
Calculated nucleation field as a function of the interface anisotropy constant *K* ^*int*^ based on Eq. (10) for Nd_2_Fe_14_B/α-Fe multilayers. (**a**) Nucleation fields for various soft layer thickness at *t* ^*h*^ = 10 nm. (**b**) Nucleation fields for various hard layer thickness at *t* ^*s*^ = 6 nm.

Figure 2 shows the calculated nucleation field *H*_*N*_ of Nd_2_Fe_14_B/α-Fe multilayers as a function of the interface anisotropy constant *K* ^*int*^ based on Eq. (10) for various values of layer thickness. The parameters for the calculation are shown in Table 1 ^31^. Nd_2_Fe_14_B is selected as the hard phase because it is the best permanent magnet so far with the largest reported energy product due to its high values for both the crystalline anisotropy and the spontaneous magnetization. On the other hand, Fe is the most abundant metal in the world, which has higher spontaneous magnetization and can be exchange-coupled with Nd_2_Fe_14_B easily. In addition, thin-film Fe can have either the positive, negative or zero surface anisotropy.

One can see from Fig. 2 that all calculated nucleation fields increase linearly with *K* ^*int*^ no matter what the layer thicknes is. Such a linear increase of the nucleation field is important, which will be addressed later in more detail. In particular, the effect of the interface anisotropy on the nucleation field is significant. For example, at *t* ^*h*^ = 10 nm and *t* ^*s*^ = 4 nm, calculated nucleation fields are 3.89 kOe, 0.13 kOe, and 7.50 kOe for *K* ^*int*^ = 0 erg/cm^2^, *K* ^*int*^ = −1 erg/cm^2^ and 1 erg/cm^2^ respectively, where *H*_*N*_ changes by more than 90%.

At a certain interface anisotropy constant *K* ^*int*^, *H*_*N*_ falls monotonically when the soft layer thickness *t* ^*s*^ goes up, as shown in Fig. 2(a). On the other hand, the nucleation field increases with *t* ^*h*^ as shown in Fig. 2(b). However, the calculated *H*_*N*_ doesn’t change when *t* ^*h*^ > 10 nm. The effect of the thickness on the nucleation field *H*_*N*_ for a hard/soft multilayer is closely related to the Bloch wall width Δ^28, 29^. When *t* ^*h*^ is large enough so that the value of the hyperbolic tangent function on the right side of the nucleation field Eq. (10) is equal to1, the nucleation field Eq. (10) can be simplified as:

11 $$\sqrt{-{A}^{s}{J}^{s}(1-\frac{{H}_{N}}{{H}_{K}^{s}})}\,\tan \,(\frac{{t}^{s}}{2}\sqrt{-\frac{{J}^{s}}{{A}^{s}}}\sqrt{1-\frac{{H}_{N}}{{H}_{K}^{s}}})=\sqrt{{A}^{h}{J}^{h}}\sqrt{1-\frac{{H}_{N}}{{H}_{K}^{h}}}+{K}^{int},$$

where ${H}_{K}^{s}=\frac{2{J}^{s}}{{M}_{S}^{s}}$ and ${H}_{K}^{h}=\frac{2{J}^{h}}{{M}_{S}^{h}}$ are the anisotropy fields of the soft and hard phases, respectively. Eq. (11) indicates that the nucleation field *H*_*N*_ is independent on the thickness *t* ^*h*^ when the hard layer is thick enough in comparison with the corresponding domain wall width.

**Figure 3:**
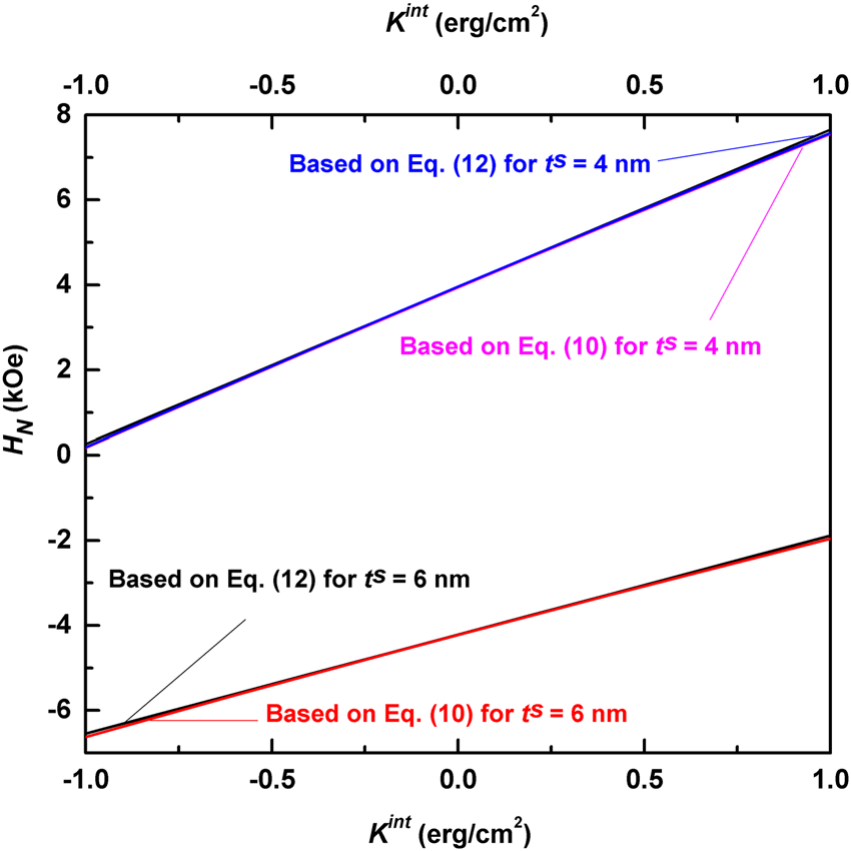
Comparison of the nucleation fields based on Eqs (10) and (12) for Nd_2_Fe_14_B/α-Fe multilayers, demonstrating excellent agreement between the two formulas. The hard layer thickness is set as 12 nm in calculating the nucleation fields from Eq. (10).

To understand more clearly the linear relation between the nucleation field and the interface anisotropy, we denote *H*_*N*_ = *H*_*N*0_ + Δ*H*_*N*_, where *H*_*N*0_ is the nucleation field based on Eq. (11) for *K* ^*int*^ = 0 erg/cm^2^. Δ*H*_*N*_ stands for the influence of the interface anisotropy on the nucleation field, which can be obtained by series expansion and simplification of Eq. (11):

12 $${\rm{\Delta }}{H}_{N}=\frac{{K}^{int}}{\frac{{t}^{s}{J}^{s}}{4{H}_{K}^{s}}+\frac{\sqrt{{A}^{h}{J}^{h}}}{2{H}_{K}^{h}\alpha }-\frac{\sqrt{{A}^{h}{J}^{h}}{\alpha }^{2}}{{H}_{K}^{s}{\beta }^{2}}(\frac{1}{2\alpha }+{t}^{s}\sqrt{{A}^{h}{J}^{h}}/4{A}^{s})},$$

where *α* = $\sqrt{1-\frac{{H}_{N0}}{{H}_{K}^{h}}}$ and *β* = $\sqrt{1-\frac{{H}_{N0}}{{H}_{K}^{s}}}$. Figure 3 compares the *K* ^*int*^-dependent nucleation fields for two values of the soft layer thickness based on Eqs (10) and (12). In both cases, the maximum differences between the nucleation fields calculated by the two formulas are less than 1.3%, indicating that the linear formula given by Eq. (12) is a very good approximation for Eq. (10).

Remarkably, such a linear approximation is valid for other hard/soft multilayers in a wide thickness range. As shown in the supplementary information, for all six combinations of hard/soft materials with various soft layer thickness, the calculated lines based on Eq. (12) are in excellent agreement with those by Eq. (10). Therefore, the nucleation fields for hard/soft multilayers can be reliably calculated by:

${H}_{N}={H}_{N0}+s\ast {K}^{int}$, where *s* is independent on the interface anisotropy. The nucleation fields at *K* ^*int*^ = 0 erg/cm^2^ and the slope *s* for various hard/soft multilayers have been shown in Table 2 for reference. One can see that *s* increases as the soft layer thickness decreases, indicating that the effect of the interface anisotropy is more evident when the soft layer thickness is small.

**Table 2:** The nucleation field at *K* ^*int*^ = 0 erg/cm^2^ (*H*_*N*0_) and the slope (*s*) based on Eq. (12) for various hard/soft multilayers.

Hard/soft (*t* ^*s*^) multilayers		4 nm	6 nm	8 nm	10 nm	12 nm
Nd_2_Fe_14_B/α-Fe	*H*_*N*0_ (kOe)	3.960	−4.216	−8.766	−11.608	−13.526
	*s* (kOe · cm^2^/erg)	3.694	2.330	1.580	1.128	0.837
Nd_2_Fe_14_B/Fe_65_Co_35_	*H*_*N*0_ (kOe)	−2.402	−10.229	−14.105	−16.928	−18.580
	*s* (kOe · cm^2^/erg)	2.994	1.730	1.094	0.737	0.520
SmCo_5_/α-Fe	*H*_*N*0_ (kOe)	14.489	1.438	−5.260	−9.247	−11.845
	*s* (kOe · cm^2^/erg)	3.696	2.117	1.349	0.920	0.658
SmCo_5_/Fe_65_Co_35_	*H*_*N*0_ (kOe)	5.163	−6.462	−12.210	−15.524	−17.624
	*s* (kOe · cm^2^/erg)	2.781	1.461	0.869	0.560	0.382
FePt/α-Fe	*H*_*N*0_ (kOe)	5.853	−3.466	−8.400	−11.406	−13.403
	*s* (kOe · cm^2^/erg)	3.936	2.390	1.595	1.129	0.834
FePt/Fe_65_Co_35_	*H*_*N*0_ (kOe)	−1.314	−9.873	−14.257	−16.861	−18.547
	*s* (kOe · cm^2^/erg)	3.113	1.751	1.066	0.736	0.519

### Microscopic hysteresis loops and angular distributions

**Figure 4:**
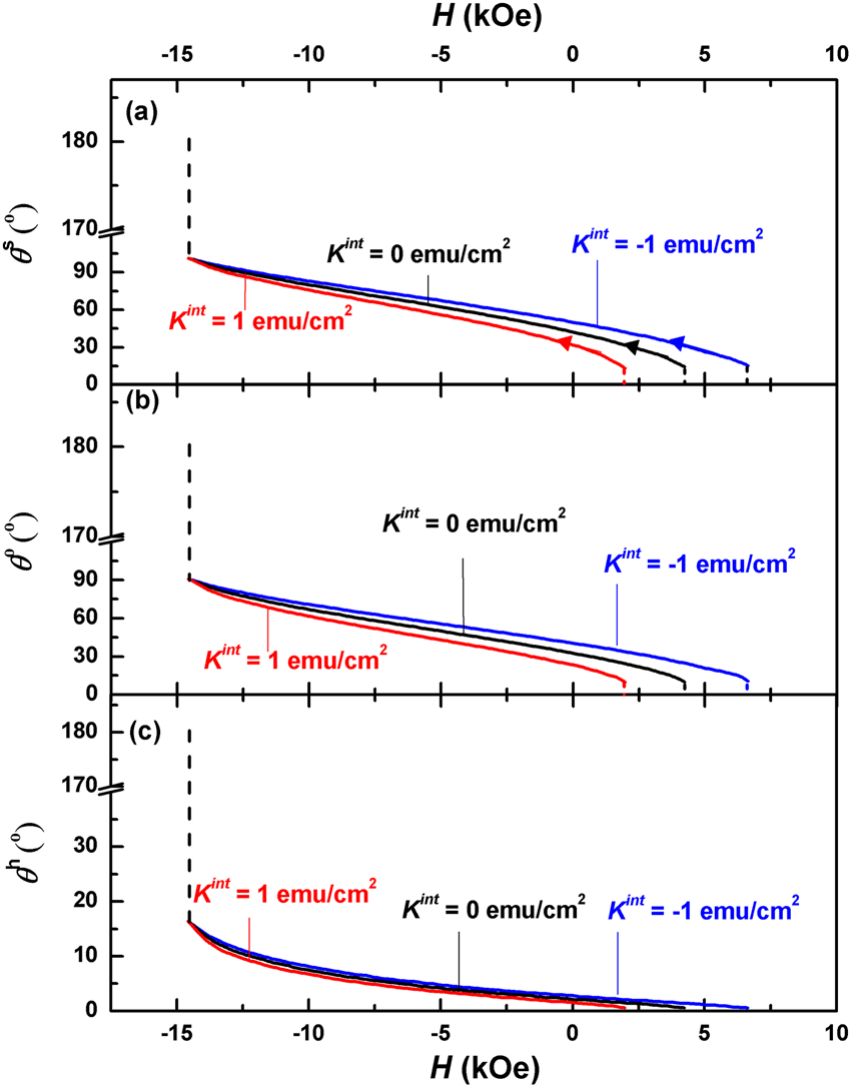
Evolution of *θ* ^*h*^ (**a**), *θ* ^0^ (**b**) and *θ* ^*s*^ (**c**) of a Nd_2_Fe_14_B (10 nm)/α-Fe (6 nm) multilayer for various interface anisotropy as the applied field changes from nucleation to pinning. The arrows in Fig. 4(a) represent the direction of demagnetization progress.

Nucleation is the beginning of the magnetic reversal, while the subsequent reversal process can be found from the microscopic hysteresis loops and angular distributions after nucleation. Figure 4 shows the evolution of three key angles, *i*.*e*., *θ* ^*h*^, *θ* ^0^ and *θ* ^*s*^ in a d_2_Fe_14_B (10 nm)/α-Fe (6 nm) multilayer with the applied fields for various interface anisotropies. All three angles deviate from 0° (the saturation magnetic state) at the different applied fields, which are *H* = 6.6 kOe, 4.2 kOe, and 2.0 kOe, for *K* ^*int*^ = −1 erg/cm^2^, 0 erg/cm^2^, and 1 erg/cm^2^ respectively, consistent with the calculated nucleation fields shown in Fig. 2. However, *θ* ^*s*^ and *θ* ^0^ are much larger than *θ* ^*h*^, indicating that the magnetic moment in the soft layer responds to the applied field fast, which draws the moments at the interface and in the hard layer to follow the response through the exchange interaction.

As the applied field decreases, *θ* ^*h*^, *θ* ^0^ and *θ* ^*s*^ increase gradually. Meanwhile, the interface anisotropy has a minor influence on the angles. As can be seen from Fig. 4, the angles for *K* ^*int*^ = −1 erg/cm^2^ are slightly larger than the corresponding ones for *K* ^*int*^ = 0, while those for *K* ^*int*^ = 1 erg/cm^2^ are slightly smaller. This minor influence disappears at *H* = −14.6 kOe, where the angles are independent of the interface anisotropy (the corresponding applied field is defined as the pinning field *H*_*P*_). Further decrease of the applied field will lead to the abrupt change of all three angles to 180°, the negative saturation state of the system.

**Figure 5:**
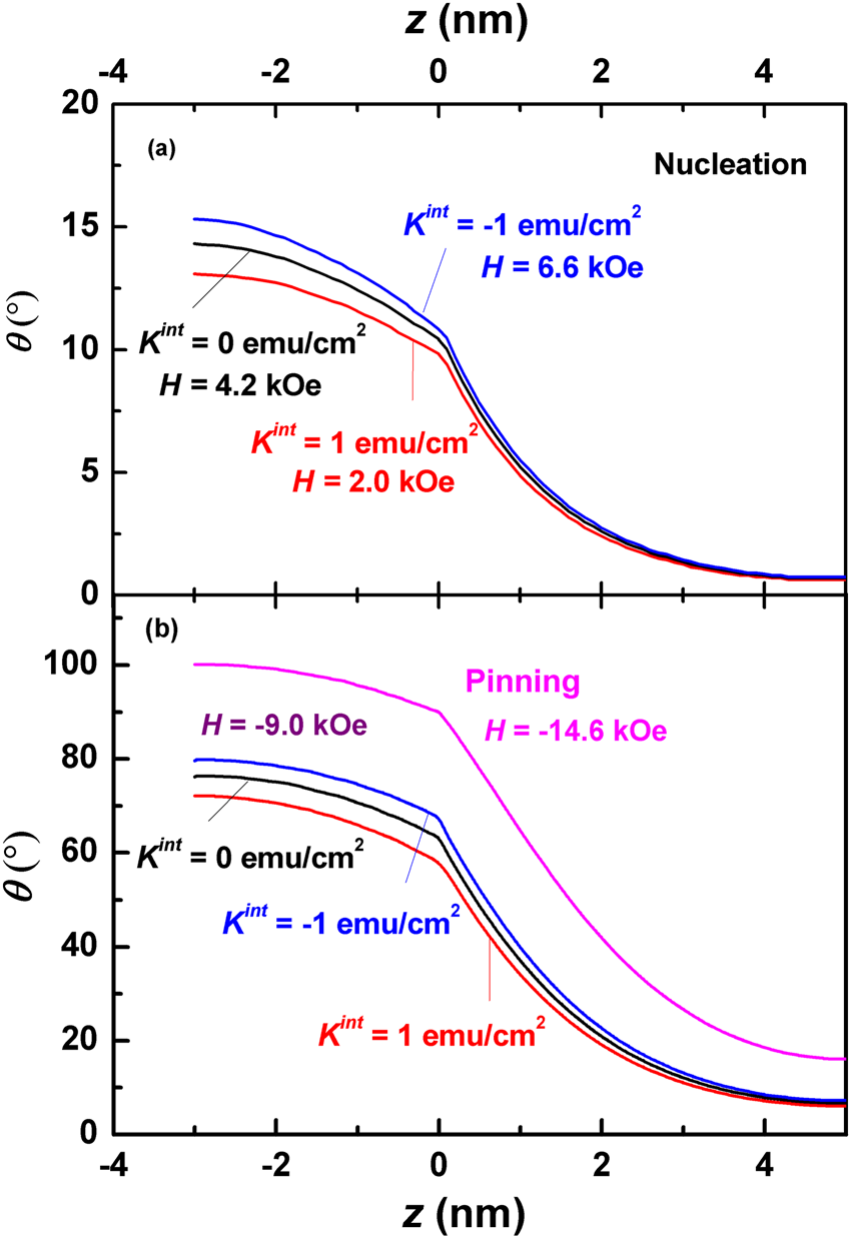
Calculated angular distributions *θ*(*z*) of the magnetizations in the thickness directions of a Nd_2_Fe_14_B (10 nm)/α-Fe (6 nm) multilayer for various interface anisotropy and applied fields. (**a**) Angular distributions at the nucleation, where the nucleation fields differ for different anisotropy constants. (**b**) angular distributions at *H* = −9.0 kOe and *H* = −14.6 kOe (the pinning). It should be noted that the three curves for various values of interface anisotropy degenerate to one single curve at the pinning.

In order to visually illustrate the change of the magnetic domain in the demagnetization process, we have calculated the angular distribution *θ*(*z*) of the magnetic moments in a Nd_2_Fe_14_B (10 nm)/α-Fe (6 nm) multilayer for various interface anisotropies at three applied fields. Due to the symmetry, only the magnetization distributions within the halves of soft and hard layers have been demonstrated (cf. Fig. 1). As can be seen from Fig. 5(a), a small deviation from the saturation state, *θ* $\equiv$ 0°, occurs at the nucleation. For *K* ^*int*^ = 0, *θ* ^*s*^ and *θ* ^0^ are 14.3°, 10.4° respectively, which are much larger than *θ* ^*h*^(=0.7°), indicating that the magnetizations of the soft layer respond to the applied field fast, which then drag the magnetizations in the hard layer to follow the change of the applied field through the exchange interaction. Therefore, a 13.6° domain wall is formed between the hard and soft layers. It should be noted that the angular change at the interface is not smooth because the first derivative of the angle θ is not continuous, as can be seen from Eq. (4). The interface anisotropy has some slight effect on the angles. A negative interface anisotropy adds a minus to the total anisotropy, leading to a faster response of the magnetizations to the applied field, which pushes the angles up a little bit. On the other hand, the angles shrink under a positive interface anisotropy, which hinders the response of the magnetizations to the applied field. As a result, the prototype domain walls forming at the nucleation are 12.5° and 14.6° for *K* ^*int*^ = 1 erg/cm^2^ and −1 erg/cm^2^ respectively.

When the applied field decreases, such prototype domain walls grow fast. As can be seen from Fig. 5(b), all three domain walls become mature at *H* = −9.0 kOe, which are 72.4°, 70.5° and 66.1° for *K* ^*int*^ = −1 erg/cm^2^, 0 erg/cm^2^ and 1 erg/cm^2^ respectively. At the pinning state, the three domain walls degenerate to a single one, where *θ* ^*h*^, *θ* ^0^ and *θ* ^*s*^ are 16.2°, 90.0° and 100.2° respectively. Further decrease of the applied field will lead to another coherent state of the system, *θ* $\equiv$ 180°.

### Macroscopic hysteresis loops and energy products

**Figure 6:**
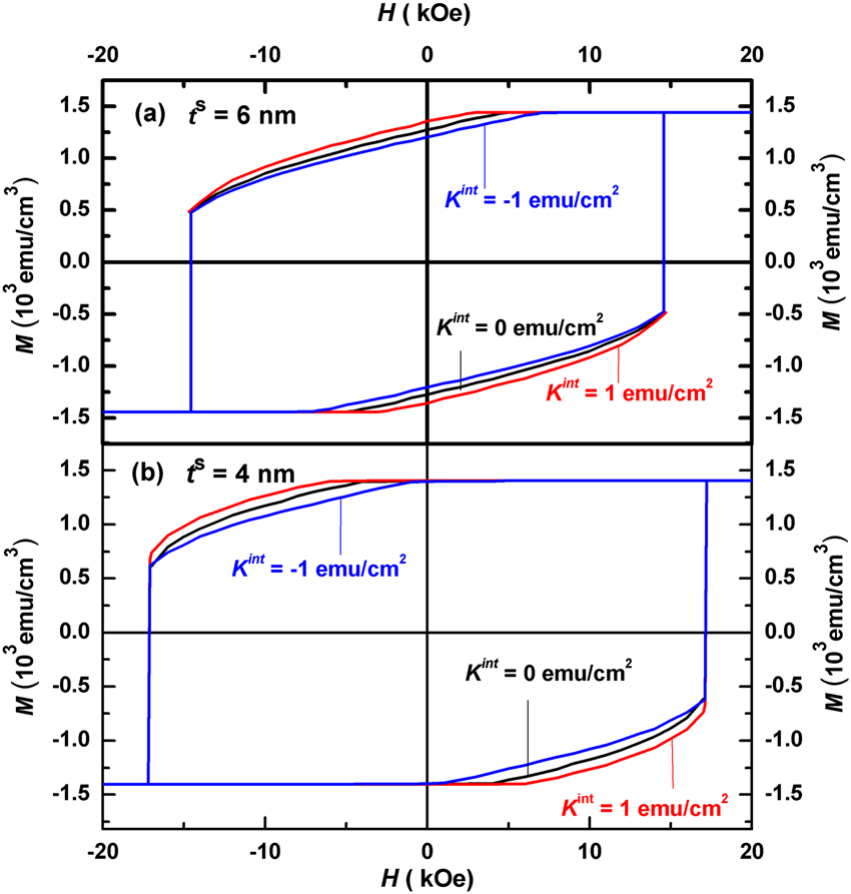
Calculated hysteresis loops of Nd_2_Fe_14_B (10 nm)/α-Fe (*t* ^*s*^) multilayers for various interface anisotropy. (**a**) Loops for soft layer thickness *t* ^*s*^ = 6 nm. (**b**) Loops for *t* ^*s*^ = 4 nm.

While the above microscopic loops and angular distributions reveal the switching mechanism well, the macroscopic hysteresis loops demonstrate explicitly the magnetic properties of the hard/soft multilayers. Figure 6 shows the calculated macroscopic hysteresis loops of the Nd_2_Fe_14_B (10 nm)/α-Fe (*t* ^*s*^) multilayers for various interface anisotropies. As shown in Fig. 6(a) for *t* ^*s*^ = 6 nm, nucleation occurs in the first quadrant, where the nucleation fields equal −2.0 kOe, −4.2 kOe and −6.6 kOe for *K* ^*int*^ = 1 erg/cm^2^, 0 erg/cm^2^, and −1 erg/cm^2^ respectively. The corresponding remanences are 1.36 × 10^3^ emu/cm^3^, 1.27 × 10^3^ emu/cm^3^ and 1.20 × 10^3^ emu/cm^3^ respectively. The greater the interface anisotropy, the larger the nucleation field is and hence the higher the remanence. On the other hand, the interface anisotropy has no effect on the coercivity, which equals to 14.6 kOe, the pinning field of the system.

In comparison, the influence of the soft layer thickness on the hysteresis loops is more visible. As can be seen from Fig. 6(b), the nucleation occurs in the second quadrant at a smaller soft layer thickness (*t* ^*s*^ = 4 nm) for all three interface anisotropy constants. Here the loops exhibit better squareness in comparison with Fig. 6(a), thanks to the larger remanence (1.40 × 10^3^ emu/cm^3^) due to the positive nucleation fields. Theoretically a decrease of the soft layer thickness can either have a positive or a negative effect on the remanence. The former is due to the larger nucleation field whilst the latter is because of the smaller saturation magnetization caused. Therefore, an optimum soft layer thickness can be found for hard/soft bilayers, as has been done in ref. 23. Further, the coercivity and the pinning field (*H*_*C*_ = *H*_*P*_ = 17.1 kOe) equal for three anisotropy interface constants, which are obviously larger than those in Fig. 6(a).

**Figure 7:**
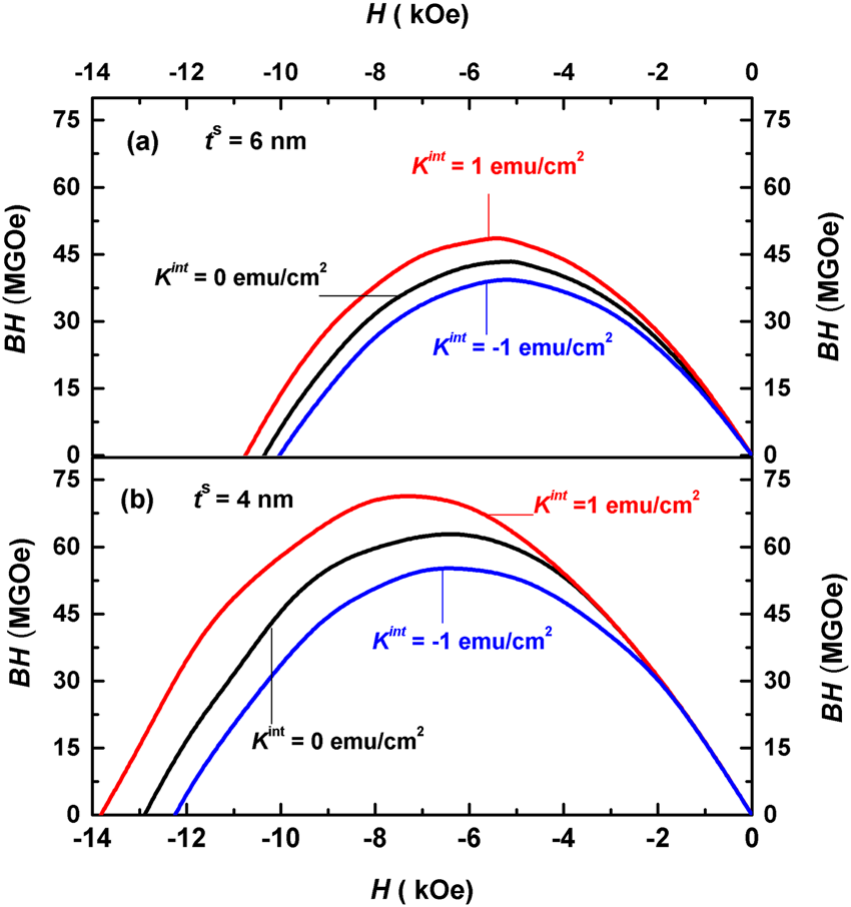
Calculated magnetic energy products (*BH*) of Nd_2_Fe_14_B (10 nm)/α-Fe (*t* ^*s*^) multilayers for various interface anisotropy. (**a**) Energy products for soft layer thickness *t* ^*s*^ = 6 nm. (**b**) energy products for *t* ^*s*^ = 4 nm.

From the macroscopic hysteresis loops, magnetic energy products (*BH*) of Nd_2_Fe_14_B (10 nm)/α-Fe (*t* ^*s*^) multilayers for various interface anisotropies can be calculated, which are shown in Fig. 7. As shown in Fig. 7(a), the maximum energy products (*BH*)_max_ of the multilayers with *t* ^*s*^ = 6 nm are 40.0 MGOe, 43.4 MGOe, and 49.0 MGOe for *K* ^*int*^ = −1 erg/cm^2^, 0 erg/cm^2^, and 1 erg/cm^2^, respectively. The increase of the (*BH*)_max_ with the interface anisotropy is due mainly to the larger remanence *M*_*r*_, caused by the larger nucleation field.

Shown in Fig. 7(b) is the calculated energy products of the multilayers for *t* ^*s*^ = 4 nm, where (*BH*)_max_ are 55.3 MGOe, 63.6 MGOe and 72.5 MGOe for *K* ^*int*^ = −1 erg/cm^2^, 0 erg/cm^2^, and 1 erg/cm^2^, respectively. These (*BH*)_max_ are considerably larger than those in Fig. 7(a). The maximum energy product (*BH*)_max_ decreases by 13% for *K* ^*int*^ = −1 erg/cm^2^ and increases by 14% when *K* ^*int*^ = 1 erg/cm^2^ relative to that of *K* ^*int*^ = 0 erg/cm^2^. The effect of the interface anisotropy on the maximum magnetic energy product (*BH*)_max_ is obvious.

## Discussions

Positive interface anisotropy enhances the nucleation field, remanence and the energy products as shown above, whilst negative anisotropy deteriorates all these magnetic properties. Therefore, it is important to have positive interface anisotropy in experiments. Experimental results suggest that negative interface anisotropy might occur when the interface of α-Fe is the 110 plane. Besides the lattice orientation, the nature of the interface and the temperature have also important influences on the interface anisotropy. More details can be found in refs 37, 41–43.

**Figure 8:**
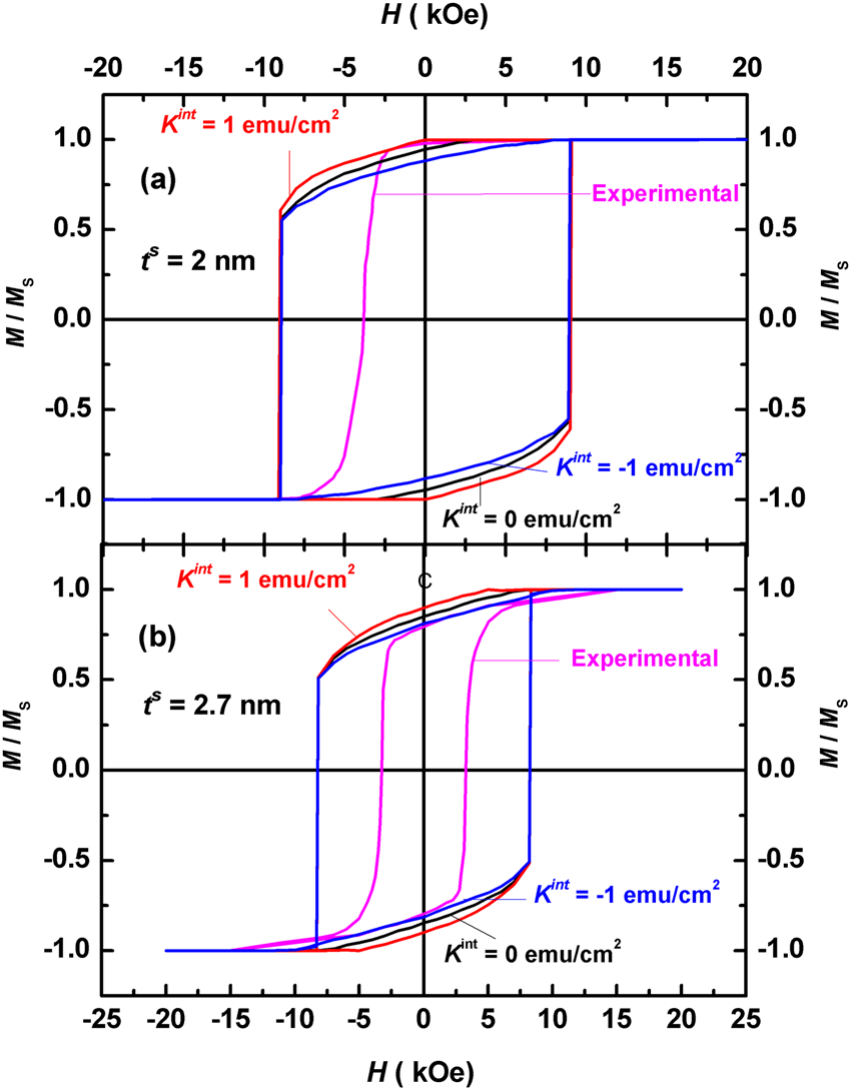
Comparison of calculated and experimental hysteresis loops of FePt (10 nm)/α-Fe (*t* ^*s*^) multilayers. (**a**) Loops for soft layer thickness *t* ^*s*^ = 2 nm, where the experimental hysteresis loop agrees well with that calculated for *K* ^*int*^ = 1 erg/cm^2^. (**b**) Loops for *t* ^*s*^ = 2.7 nm, where the experimental loop agrees well with that calculated for *K* ^*int*^ = −1 erg/cm^2^.

Figure 8 compares experimental^23^ and calculated hysteresis loops of FePt (10 nm)/α-Fe (*t* ^*s*^) bilayers. One can see from Fig. 8(a) that the experimental hysteresis loop near the remanence for *t* ^*s*^ = 2 nm agrees well with that calculated for *K* ^*int*^ = 1 erg/cm^2^, exhibiting good squareness and high remanence. In particular, the nucleation field and reduced remanence measured in the experiment are −2.01 kOe and 0.98 respectively^23^. Both of them are very close to our calculated values for *K* ^*int*^ = 1 erg/cm^2^, which are −2.0 kOe and 0.99 respectively.

In contrast, the experimental loop near the remanence shown in Fig. 8(b) for *t* ^*s*^ = 2.7 nm matches well with that calculated for *K* ^*int*^ = −1 erg/cm^2^, which is more slant and exhibits lower remanence in comparison with the corresponding loop in Fig. 8(a). Here, *M*_*r*_/*M*_*S*_ = 0.89, 0.85 and 0.80 for *K* ^*int*^ = 1 erg/cm^2^, 0 and −1 erg/cm^2^ respectively according to our calculation, whilst the reduced remanence measured in the experiment is 0.79^23^. Therefore, negative interface anisotropy should be avoided in the experiment, which leads to lower nucleation fields and remanence.

The values of the experimental coercivity in both cases, however, are much smaller than the calculated ones, due to the famous Brown’s coercivity paradox^39, 40^, where the measured coercivity is found to be systematically smaller than the theoretical one for all magnetic materials. This paradox is more outstanding for a single-phased permanent magnet. As have been shown in ref. 23, the experimental coercivity for a single FePt film is only slightly larger than those for hard-soft bilayers. In contrast, the theoretical coercivity for a single FePt equals the effective anisotropy of the FePt film, *i*.*e*, the sum of the crystalline anisotropy and the shape anisotropy, which is 93.6 kOe. Such a huge difference between the theoretical and experimental values of is due possibly to the various crystalline defects within the film.

## Conclusions

In conclusion, the demagnetization process for a hard/soft multilayer with a perpendicularly oriented anisotropy is analyzed by a one-dimensional micromagnetic model, where both positive and negative interface anisotropies have been taken account into the calculation. It is found that the nucleation field and the remanence are sensitive to *K* ^*int*^, especially for smaller soft layer thickness. On the other hand, the interface anisotropy doesn’t affect the angular distributions much, which has no effect on the coercivity. Our calculated macroscopic hysteresis loop and energy product show that a positive *K* ^*int*^ increases the remanence or nucleation field and hence enhances the energy product, and that a negative *K* ^*int*^ deteriorate both of them. Comparison with experimental data indicates that the interface anisotropy can be either positive or negative, where a positive *K* ^*int*^ is preferred for a high remanence and a large energy product.

Further, the formula for the nucleation field is derived analytically, which is found to be linearly related to the interface anisotropy for all calculated values of layer thickness. Therefore, a simplified linear formula for the nucleation field is educed, which is reliable for all combinations of the hard/soft layers calculated. The influence of the interface anisotropy is more significant for smaller soft layer thickness, where the interface anisotropy can change the nucleation field by more than 90% in comparison with that for *K* ^*int*^ = 0. In addition, the microscopic hysteresis loops for *θ* ^*h*^, *θ* ^0^ and *θ* ^*s*^ as well as the angular distributions in the thickness direction are calculated numerically, which shows that the effect of the interface anisotropy on the angles is not significant. In particular, the angular distributions are the same for different values of the interface anisotropy at the pinning field.

In summary, neither the coercivity nor the angular distributions of exchange-coupled magnets are sensitive to the interface anisotropy. However, the nucleation field and the energy product are quite sensitive to the interface anisotropy, especially for small soft layer thickness. The positive interface anisotropy enhances the nucleation field, the remanence and the energy product, whereas the negative anisotropy deteriorates all these properties. Therefore, it is important to have the positive interface anisotropy in experiments, where small layer thickness is preferred for a giant energy product to be realized in exchange-coupled multilayers.

## Electronic supplementary material

Supplementary Information
