# Supplementary material for: The effect of interface anisotropy on demagnetization progress in perpendicularly oriented hard/soft exchange-coupled multilayers
Source: Sci Rep. 2017 Jun 27;7:4286. doi: 10.1038/s41598-017-03169-y (PMC5487335; doi:10.1038/s41598-017-03169-y)
Supplement: Supplementary file 1 — Supplementary Information [file 41598_2017_3169_MOESM1_ESM.pdf]

The effect of interface anisotropy on demagnetization progress in perpendicularly oriented hard/soft exchange-coupled multilayers

Qian Zhao[1], Jun Chen[1], Jiaqi Wang[1], Xuefeng Zhang[1]*, Guoping Zhao[1,2] & Qiang Ma[1]

[1]Inner Mongolia Key Laboratory for Utilization of Bayan Obo Multi-Metallic Resources: Elected State Key Laboratory, Inner Mongolia University of Science and Technology, Baotou 014010, China

Department of Applied Physics, College of Science, Inner Mongolia University of Science and Technology, Baotou 014010, China

[2]College of Physics and Electronic Engineering, Sichuan Normal University, Chengdu, 610066, China

*correspondence to xuefeng056@163.com

SUPPLEMENTARY INFORMATION

Supplementary Note 1: Calculated K^{int}-dependent nucleation fields of various hard/soft multilayers for five values of soft layer thickness based on Eqs. (10) and (12)

In Fig 3, we have demonstrated that there is a linear relation between the nucleation field and the interface anisotropy for $Nd_2Fe_{14}B$ (12 nm) /α-Fe multilayers with two values of the soft layer thickness. In addition, Eq. (10) can be simplified to Eq. (12) reliably. To find whether such a linear relation and the related simplification are general, we have calculated the K^{int}-dependent nucleation fields of various multilayers for five values of the soft layer thickness based on Eqs. (10) and (12), which are shown in Figs. S1 - S6. Good linear relationship sustains for all the lines calculated in the six figures, indicating that the linear relationship is general. Moreover, in each case, the calculated line based on Eq. (12) coincides with that by Eq. (10), demonstrating the validity of Eq. (12) in a wide thickness region, which suits most hard/soft materials.

Relatively larger error occurs in Figs. 5 and 6 due to the comparatively larger

domain wall width, where FePt is used as the hard phase. One can see from Table 1 that the Bloch wall width of FePt is 7.02 nm [1], which is comparable to its thickness (12 nm) adopted in the calculation. As has been shown in the main text, Eq. (10) can be simplified to Eq. (11) only when the hard layer thickness is much larger than the corresponding Bloch wall width.

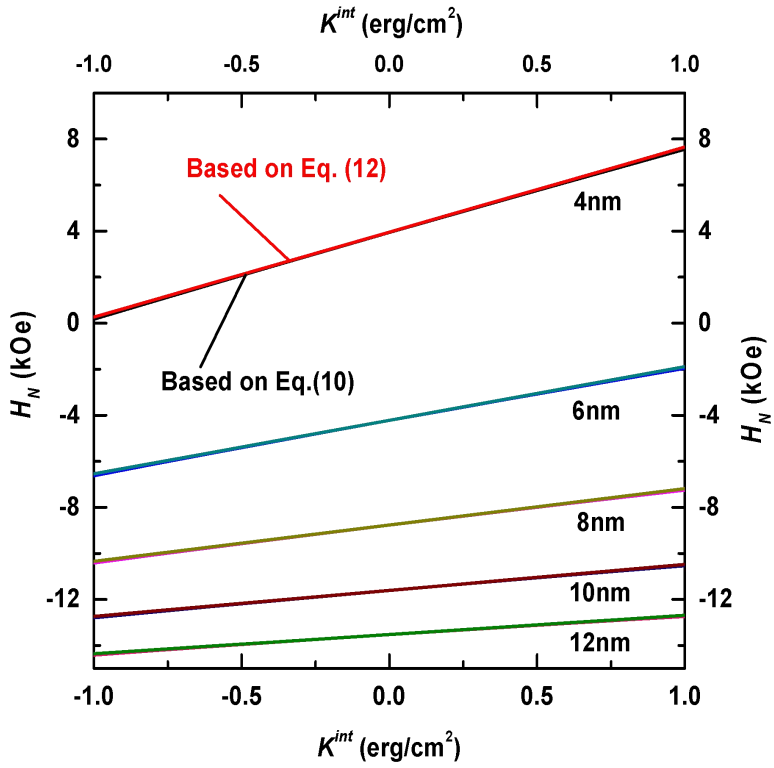

Fig. S1. Calculated K^{int}-dependent nucleation fields of $Nd_2Fe_{14}B$ (12 nm) /α-Fe (t^s) multilayers for various values of soft layer thickness based on Eqs. (10) and (12).

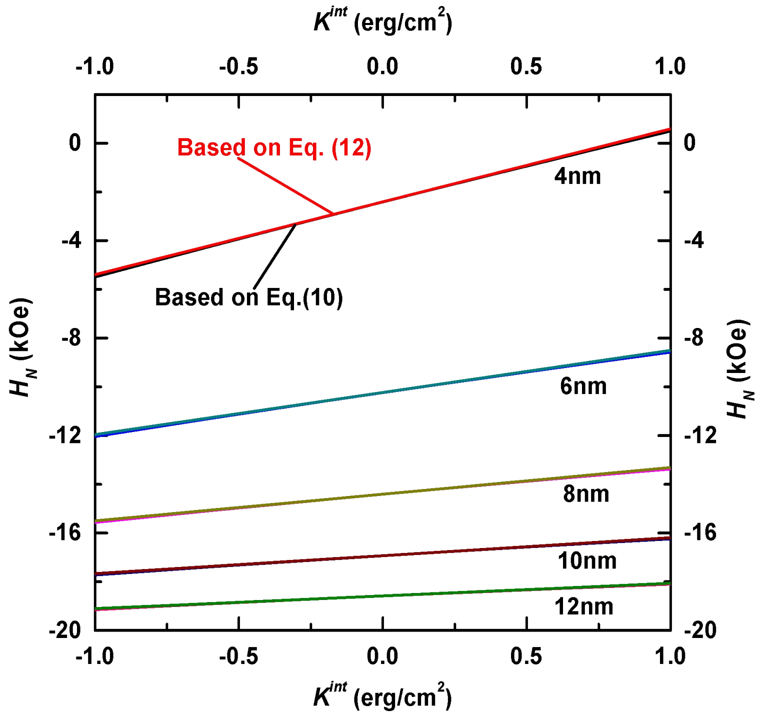

Fig. S2. Calculated K^{int}-dependent nucleation fields of $Nd_2Fe_{14}B$ (12 nm) / $Fe_{65}Co_{35}$ (t^s) multilayers for various values of soft layer thickness based on Eqs. (10) and (12).

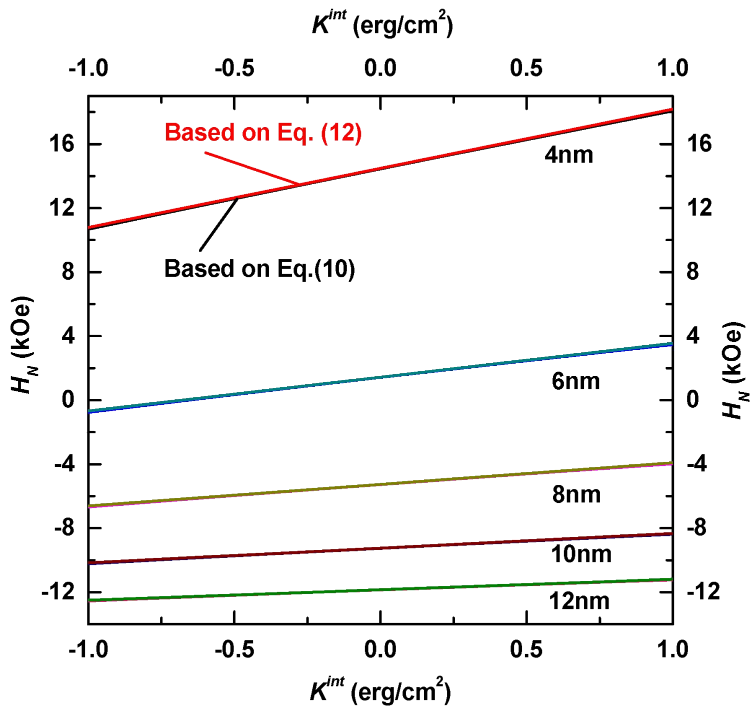

Fig. S3. Calculated K^{int}-dependent nucleation fields of $SmCo_5$ (12 nm)/α-Fe (t^s) multilayers for various values of soft layer thickness based on Eqs. (10) and (12).

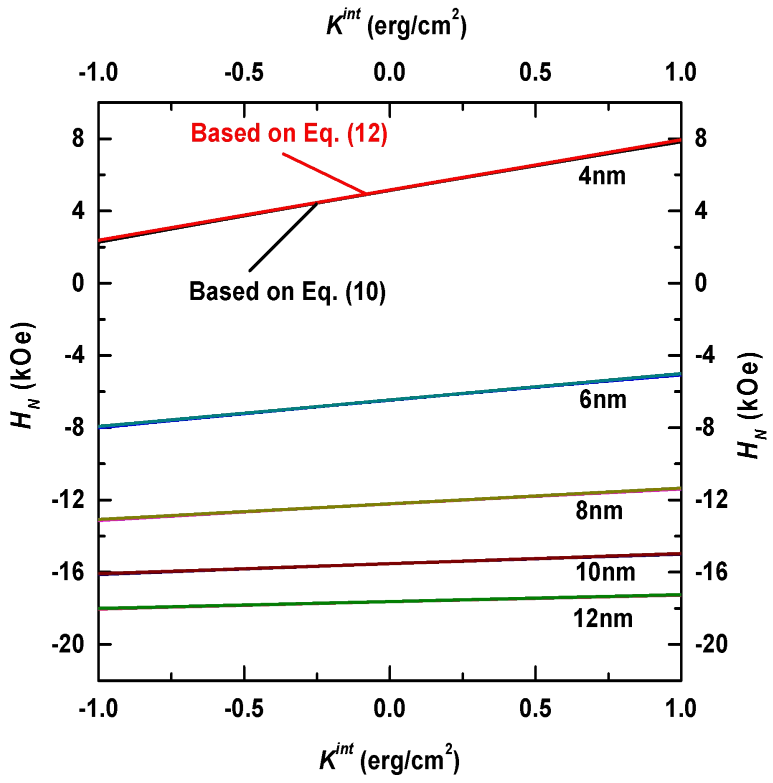

Fig. S4. Calculated K^{int}-dependent nucleation fields of $SmCo_5$ (12 nm)/$Fe_{65}Co_{35}$ (t^s) multilayers for various values of soft layer thickness based on Eqs. (10) and (12).

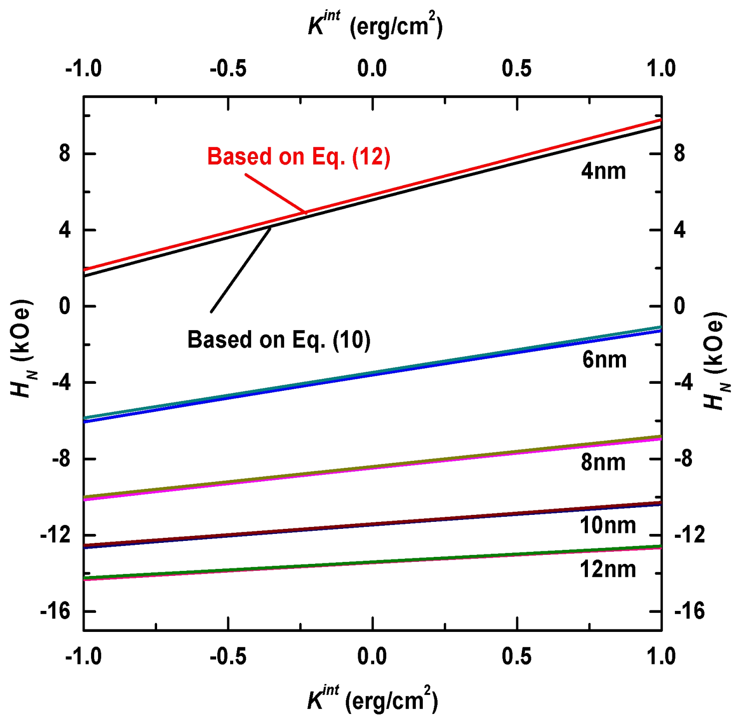

Fig. S5. Calculated K^{int}-dependent nucleation fields of FePt (12 nm)/α-Fe (t^s) multilayers for various values of soft layer thickness based on Eqs. (10) and (12).

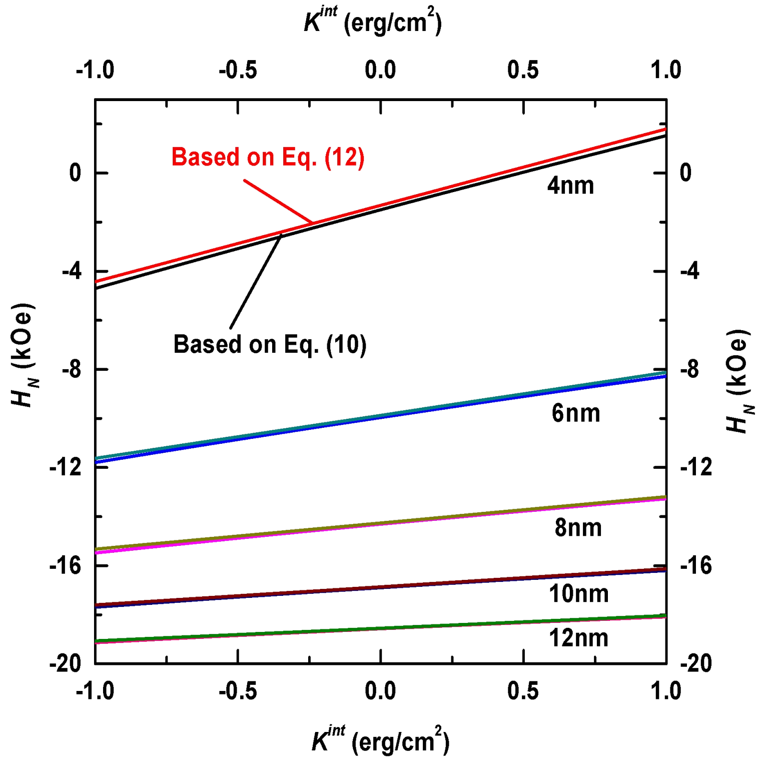

Fig. S6. Calculated K^{int}-dependent nucleation fields of FePt (12 nm)/$Fe_{65}Co_{35}$ (t^s) multilayers for various values of soft layer thickness based on Eqs. (10) and (12).

Reference in Supplementary

1. Si, W. J. *et al*. Deterioration of the coercivity due to the diffusion induced interface layer in hard/soft multilayers. *Sci. Rep.* 5, 16212 (2015).
